# Characteristics of ultrafine-ground potassium-bearing shale and the effects of roasting pre-cracking

**DOI:** 10.1038/s41598-021-02983-9

**Published:** 2022-01-10

**Authors:** Zaisheng Zhu, Zhenquan He, Guosheng Gai

**Affiliations:** 1 School of Materials Science and Engineering, Anhui University of Science and Technology, 168# Taifeng Road, Huainan, 232001 Anhui China; 2 School of Materials Science and Engineering, Tsinghua University, 30# Shuangqing Road, Beijing, 100084 Beijing China; 3 Wuxi Research Institute of Applied Technologies Tsinghua University, 777# West Jianzhu Road, Wuxi, 214100 Jiangsu China

**Keywords:** Solid Earth sciences, Mineralogy

## Abstract

Potassium-bearing shale is being developed as a potential alternative to potash for use in fertilisers. The first step in this process is to reduce its particle size by crushing. This paper explores whether roasting pre-cracked potassium-bearing shale can improve the quality of the resulting ultrafine product. Analysis of the particle size distribution of the ultrafine product and its fractal dimension found contradictory results: the minimum particle size distribution was obtained by roasting for 2.5 h, while the minimum fractal dimension was obtained by roasting for 1 h. Fuzzy comprehensive evaluation was conducted with three indicators—(1) the weight of the − 10 μm product, (2) the fractal dimension of the particle size distribution, and (3) *d*_97_—to obtain a unique combination of indicators that reflects the quality and quantity of the products. The weights of the three indicators were calculated by an analytic hierarchical process to be 0.69, 0.149 and 0.161, respectively. Roasting pre-cracked shale for 2–2.5 h was found to improve the mean values of the fuzzy comprehensive evaluation indicators by about 0.07. However, the cost increased from 2.82 RMB to ≥ 10.08 RMB, which is not feasible for widespread industrial implementation.

## Introduction

Potassium-bearing shale is a type of layered sedimentary rock that forms exploitable deposits of water-insoluble potassium-bearing rock. There are large potassium-bearing shale resources in some parts of China, such as Henan, Guizhou, Sichuan, Hubei and Hunan Province^1^. Potassium-bearing shale can be prepared into potassium sulfate^2^ and used to improve cement. It can produce low-alkali cement when blended with fly ash, which is an alternative to using clay ingredients^3^. China is a largely agricultural country, and potassium-bearing shale is a potential source of potash fertiliser until large quantities of soluble potash are found. Hence, there is a need to enhance the integrated use of potash-bearing rocks. Some researchers have sought to improve the utilization of potassium-bearing shale by adding chemicals during roasting or by applying chemical methods after roasting; however, both pose potential environmental hazards^4,5^. Potassium-bearing shale has been used to prepare potash fertilisers by physical methods^6^, with experimental research showing that the smaller the particle size of the shale, the greater its efficacy.

The micro-crack theory proposed by Griffiths suggests that there are many defects and cracks in materials^7^ which, under the action of external forces, produce stress concentrations. Beyond a certain stress level, the cracks begin to expand and form fractures, or internal microcracks grow and create many new cracks simultaneously.

Internal structural changes in rock can be attributed to a variety of factors, such as differences in the expansion coefficients of its mineral components, structural pre-cracking that occurs during roasting treatment, growth of microscopic internal fractures, pores and micropores, residual microscopic defects after water loss, and uneven stresses caused by structural thermal stresses^8^. Roasting treatment can reduce the strength of rock as well as induce grain boundary fractures, causing local stress concentrations that lead to comminution^9,10^. Since the twentieth century, studies of thermally-assisted crushing of a variety of geological minerals have shown exceptional results^11^, with some indicating that potassium-bearing shale roasting leads to acid leaching during potassium extraction^12,13^. To date, researchers have seldom reported the effects of roasting pre-cracking on potassium-bearing shales.

## Materials and methods

### Materials

**Figure 1:**
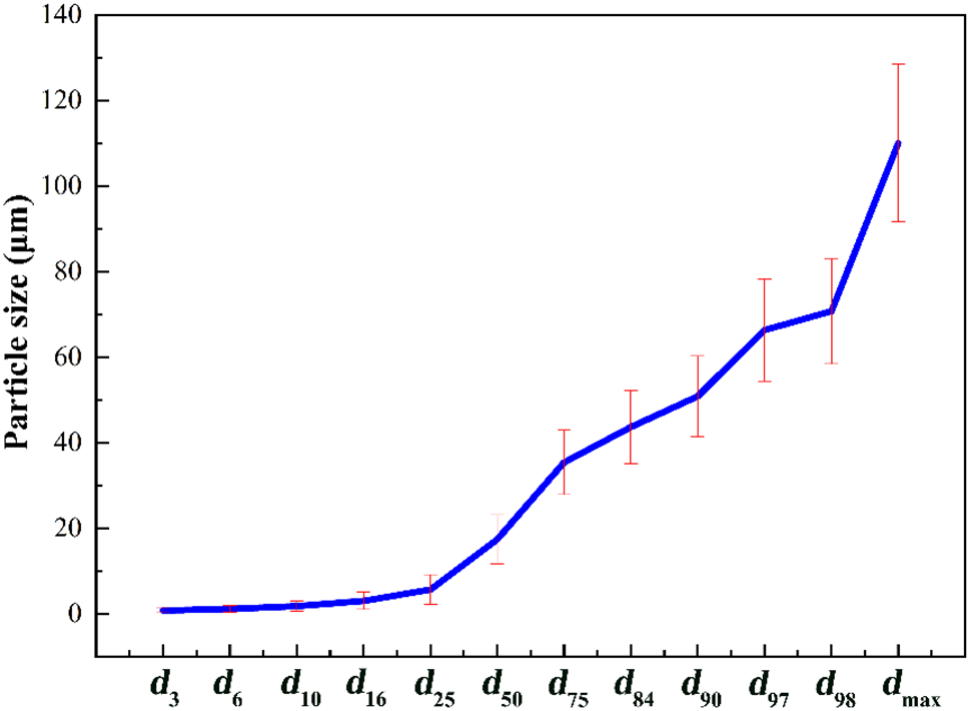
Particle size distribution of raw shale. The error bars represent the standard deviation of three measurement data, the same below.

**Figure 2:**
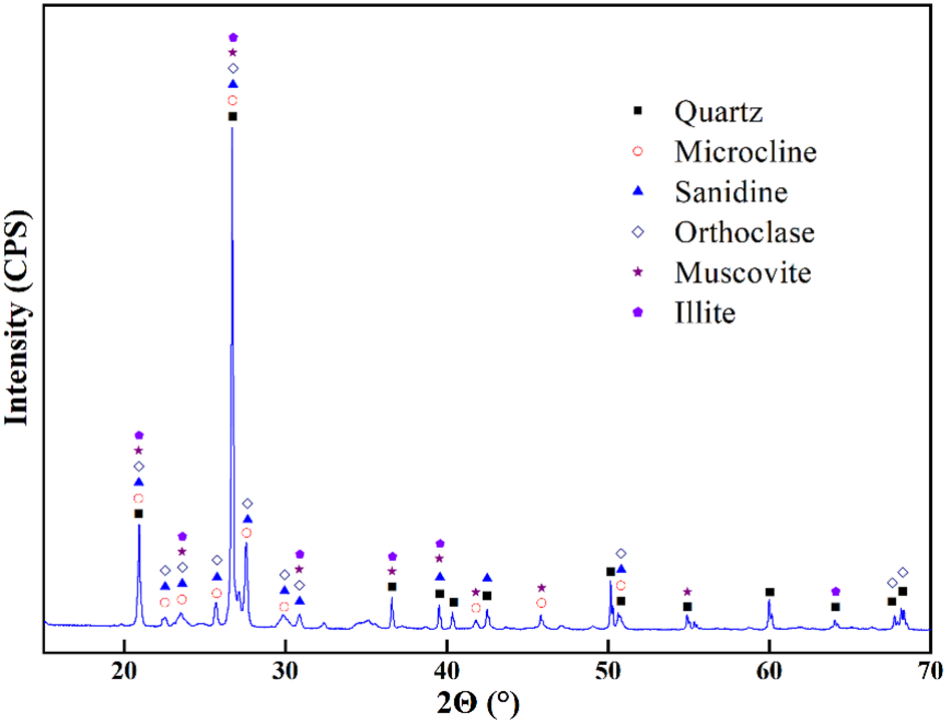
The XRD spectrum of the raw materials.

Raw potassium-bearing shale was collected from a site in Jiangsu Province and the specific surface area was measured by a digital Blaine permeability surface area analyser (SBT-127, Wuxi Jian Yi Instrument Co., Ltd.) at 141.9 m^2^/kg with a moisture content of 0.5%. The content of K_2_O was 9.07%. A laser particle size analyser (BT-9300H, Dandong Baxter Instruments Co., Ltd.) was used to detect the particle size distribution. The raw potassium-bearing shale was mixed well in a water medium, from which three samples were taken and tested. The results are shown in Fig. 1. The crystal structure was analysed by an X-ray diffractometer (Smartlab SE, Rigku, Japan) with a scanning range of 2Θ = 10–70°, operating current of 30.0 mA, operating voltage of 40.0 kV, maximum power of 12 kW, scanning speed of 5°/min in continuous mode, and a Cu Kα X-ray source. As shown in Fig. 2, the main mineral phases of the potassium-bearing shale were quartz, microcline, sanidine, orthoclase, muscovite and illite. The sharpness of each peak indicates that the mineral phases were crystalline and had small grains with good crystallinity.

### Equipment

The intelligent fibre chamber resistance furnace was a model SX3-5-12A (Tianjin Zhonghuan Experimental Electric Furnace Co. Ltd.) with a power rating of 4000 W and temperature rating of 1200 °C.

The ball mill (model QM-5, Changsha Tianchuang Powder Technology Co., Ltd.) had a power rating of 1000 W. It had a stainless steel tank with an inner diameter of 230 mm, inner height of 260 mm, volume of 10.8 L and critical speed of 88.4 RPM. The grinding media were tungsten carbide high-energy balls with a total weight of 18.52 kg (10.61 kg of 15 mm balls and 8.06 kg of 10.5 mm balls) and a specific surface area of 2.486 m^2^/kg.

### Experimental method

Some 0.957 kg of water and 1.445 kg of potassium-bearing shale at a 60% concentration were mixed, to which 2.89 g of NP-45 dispersant at 0.2% by mass was added. The tank speed was set at 80% of the critical speed, i.e., 70.72 RPM. The roasting pre-cracking method was carried out as follows: 1.445 kg of potassium-bearing shale was placed in a tray and placed in a resistance furnace at 500 °C. After roasting for 0.5–2.5 h, the potassium-bearing shale was taken out and poured directly into a ball mill jar containing equal amounts of water and grinding media, where the weight of the water included the water evaporated from the potassium-bearing shale, i.e., 0.964 kg. Natural cooling was allowed and a grinding aid was added. The shale was then ground into an ultrafine powder in the high-energy ball mill. Samples were taken at three points on the slurry surface of the ball mill jar and the particle size distribution was checked by a laser particle size analyser three times.

### AHP-fuzzy comprehensive evaluation method

An analytic hierarchical process (AHP) was used to obtain the weight vector of each indicator^14^ and fuzzy mathematics was used to calculate the comprehensive evaluation index, which transforms the multi-indicator decision problem into a single-indicator decision problem^15^. It adopts membership degree theory to assess its level and has the characteristics of clear, robust results, providing a powerful basis for the combination of qualitative and quantitative decision-making^16^.

Three indicators were selected to reflect the product’s comprehensive characteristics. In terms of measuring yield, the weight of the − 10 μm product was chosen. In terms of product quality, the fractal dimension of the particle size distribution (which indicates its width) and *d*_97_ were chosen as measures. Since only a few parameters were selected for the characterisation of yield and quality, a comprehensive evaluation system was constructed.

**Figure 3:**
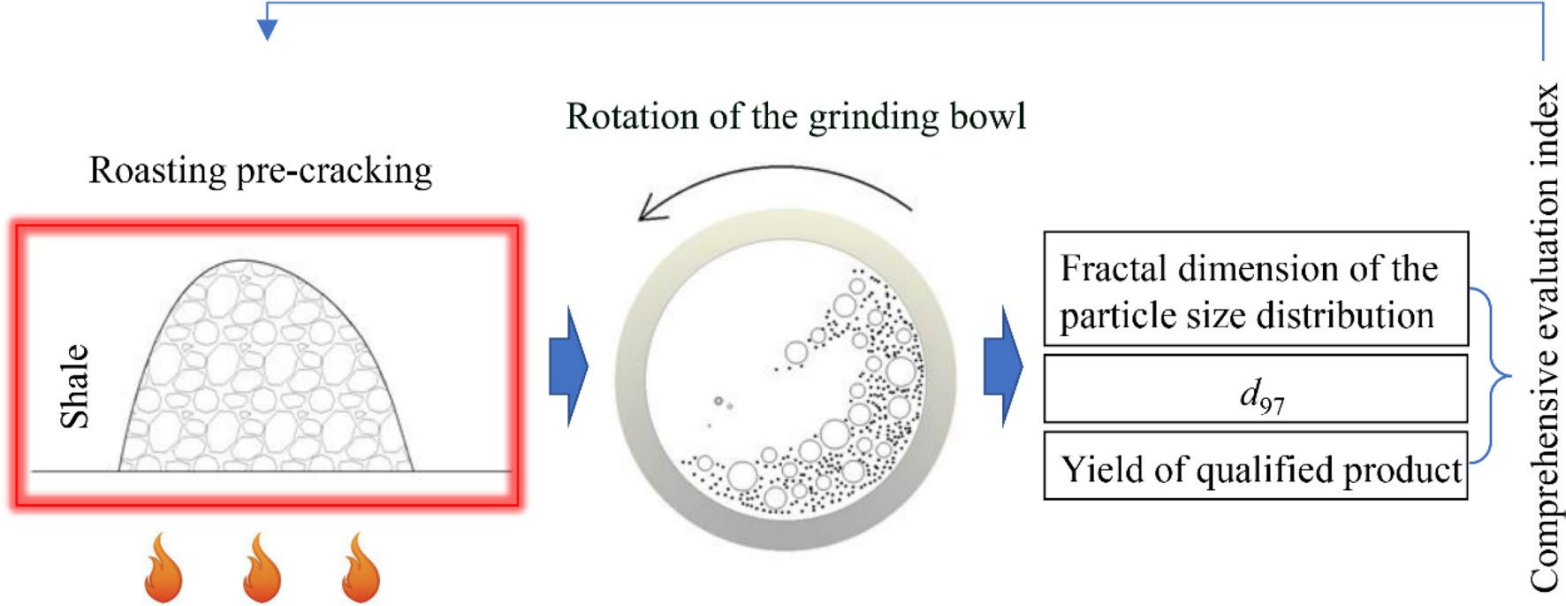
Experimental flowchart.

The experimental flow is shown in Fig. 3.

## Results and discussion

### Effect of roasting pre-crushing on particle size

**Figure 4:**
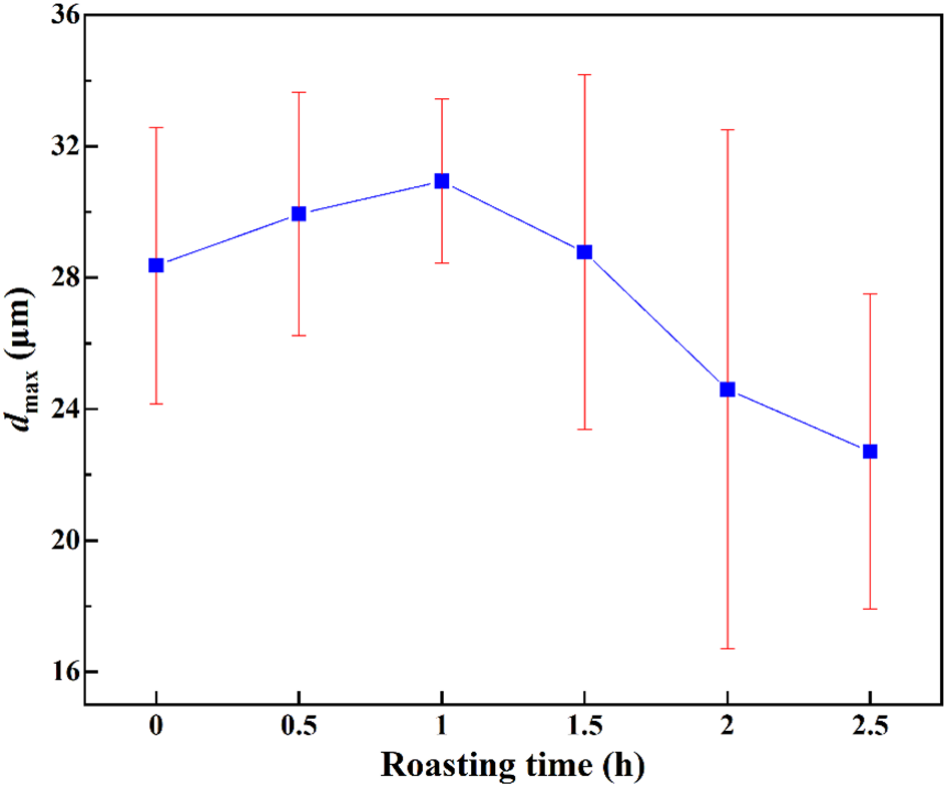
*d*_max_-values of ultrafine products made by different processing modes.

The change in the maximum particle size (d_max_) of the product is shown in Fig. 4. With increasing roasting time, *d*_max_ first increased in the first 1 h and then gradually decreased. It can be seen that roasting for 2–2.5 h and then crushing reduced the *d*_max_ value of the blank test product. Individual indicators, however, do not accurately reflect the overall quality of the product.

The crystal structures of shelf-structured potassium feldspar and layered mica-like minerals are relatively stable. Roasting increases the internal porosity of potassium-bearing shale but also increases the strength of its particles^17^. More pores are of benefit to comminution, while a higher strength is not. Roasting pre-cracking for 0.5–1.5 h may increase the pore number to a lesser degree but the powder strength increases, which increases the ultrafine product size. Roasting pre-cracking for 2 h increases pore numbers while *d*_max_ decreases.

**Figure 5:**
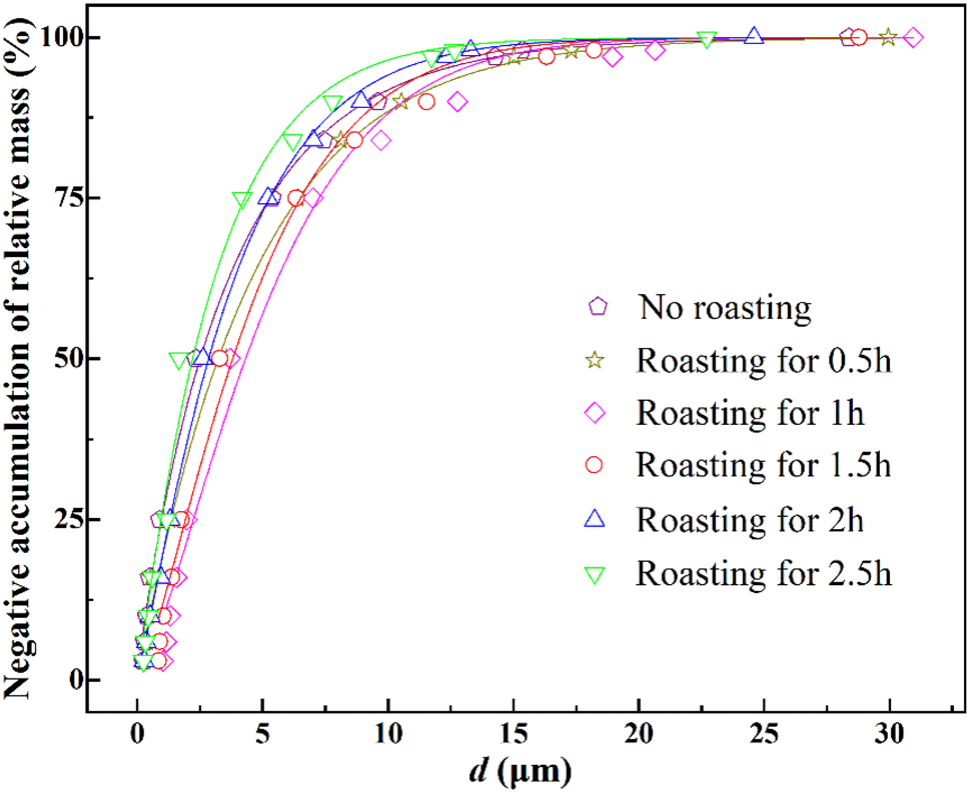
Particle size distributions of different products.

The Rosin–Rammler-Bennett distribution (RRB) is one of the most widely applied distribution patterns^18,19^, as expressed by Eq. (1).

1 $$R=100-100\cdot exp\left[-{\left(\frac{d}{{d}_{e}}\right)}^{n}\right]$$

where *d* is the particle size (μm), *R* is the negative accumulation of relative mass under a sieve of size *d* (%), *d*_e_ is the characteristic particle size corresponding to the particle size with an oversize percentage of 100–100e^-1^, i.e., 63.21% (μm), and *n* is the uniformity factor (dimensionless). Higher *n* values indicate narrower particle size distributions. The particle size distributions of both the raw potassium-bearing shale and ultrafine product conformed to the RRB model, as shown in Fig. 5 and Table 1.

**Table 1:** RRB model of products made via pre-roasting.

Roasting time (h)	*d*_*e*_ (μm)	*n* (–)	*R*^2^
0	3.73	0.93	0.99
0.5	4.66	1.01	0.99
1	5.68	1.34	0.99
1.5	5.06	1.29	0.99
2	3.95	1.12	0.99
2.5	3.14	1.04	0.99

These results show that the *d*_e_-value increased and then decreased with roasting time. The *d*_e_-value of the crushed product became smaller than that of the raw shale after roasting pre-cracking for 2.5 h, which further indicates that roasting for a short time is not conducive to ultrafine comminution. The particle size distribution became narrower and then wider with roasting time and all were narrower than those of blank test products. Detailed analysis of the particle size distribution data shows that the content of fine particles decreased after roasting which, in turn, leads to a narrower particle size distribution.

### Effect of roasting pre-cracking on the fractal dimension

The geometrical characteristics of many materials as they evolve from microscopic damage to macroscopic crushing have good statistical self-similarity, as do the mathematical characteristics of the evolution in mechanical and physical quantities^20^. This self-similar behaviour means that the particle size distribution of the crushed powder has fractal characteristics. The fractal dimension has been demonstrated in many papers to characterise the width of the particle size distribution^21,22^, and does so more accurately than the uniformity factor^23^. The higher the fractal dimension, the wider the distribution, and vice versa.

Provided that the particle size distribution conforms to the RRB model, the formula for calculating the fractal dimension of the particle size distribution is^23^:

2 $$S\left(d\right)=k\cdot {\left(\frac{d}{{d}_{\text{max}}}\right)}^{3-D}$$

where *S*(*d*) is the mass percentage under the sieve smaller than the particle size *d* (%), *D* is the fractal dimension of the particle size distribution (dimensionless), *k* is a constant, *d* is the particle size (µm) and *d*_max_ is the maximum particle size (µm).

**Figure 6:**
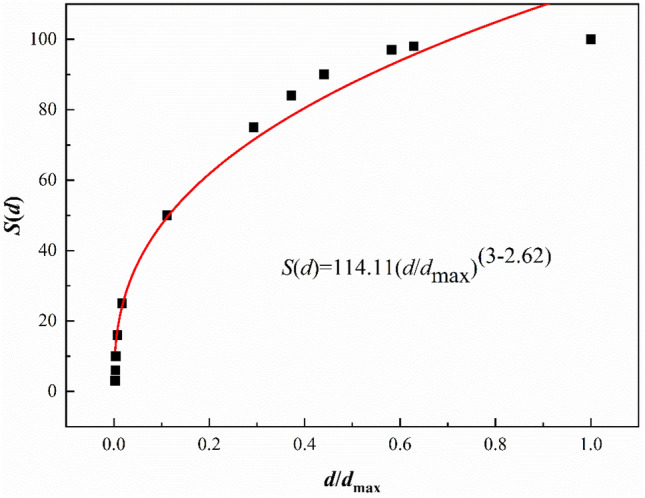
Calculation of the fractal dimension of the particle size distribution.

The *D*-value of the potassium-bearing shale feedstock was calculated in Origin 2018 by fitting the data with Eq. (2). The fractal dimension of the feedstock was *D* = 2.62 with a correlation coefficient of 0.97, as shown in Fig. 6.

**Figure 7:**
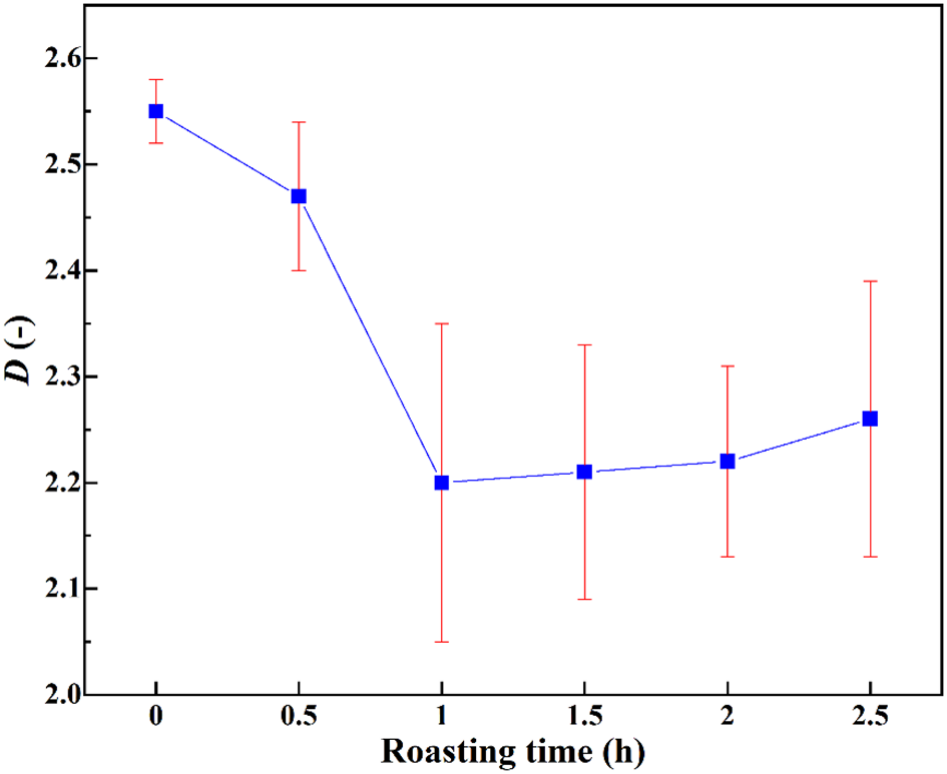
Fractal dimension of the particle size distribution of different ultrafine products.

By analogy, the *D*-values of other products were calculated. The variations in the *D*-values of the ultrafine pulverised products after different roasting pre-cracking times are shown in Fig. 7.

The yield of fine particles decreased after roasting. As can be seen from Fig. 6, the fractal dimensions of the particle size distributions of the ultrafine pulverised products were smaller than that of the raw shale. The product particle size distribution was narrowest after roasting pre-cracking for 1 h.

### Influence of roasting pre-cracking on the product according to fuzzy comprehensive evaluation

First of all, we calculated the weight vectors of the evaluation indicators. The three indicators to be evaluated were the yield of qualified product (W1), the fractal dimension of the particle size distribution (W2) and *d*_97_ (W3), where the weight vector is denoted as W.

An expert in the powder industry (Professor Gai Guosheng, Tsinghua University) compared the three indicators to be evaluated according to the relevant diagram^8^. The results of the two comparisons provided by the experts are: (I) the yield of qualified product is slightly but not significantly more important than the fractal dimension of the particle size distribution, which is significantly more important than *d*_97_, and (II) the fractal dimension of the particle size distribution is equally as important as *d*_97_.

A description of the experts’ combined comparison of the indicators can be expressed as a descriptive matrix of importance and then transformed into a judgment matrix, ${A}_{3\times 3}$.

$${A}_{3\times 3}=({a}_{\text{ij}})_{3\times 3}=\left[\begin{array}{ccc}1& 5& 4\\ 0.2& 1& 1\\ 0.25& 1& 1\end{array}\right]$$

The weight vector is calculated as $W={\left({0.69,0.149,0.161}\right)}^{T}$.

Secondly, a fuzzy comprehensive evaluation system must be established.

(1) Determining the evaluation set of test results. For the blank test, the three measurement results of the product after 3 h of ultrafine grinding are shown in Table 2.

**Table 2:** Three times parallel values of product indicators used in the blank test.

No	W1 (kg)	W2 (–)	W3 (μm)
1	1.31	2.54	14.27
2	1.29	2.52	16.83
3	1.36	2.58	12.69

The evaluation sets of the three measurement results are: U = {W1,W2,W3} = {1.31,2.54,14.27}, {1.29,2.52,16.83}, {1.36,2.58,12.69}.

(2) Determining the value ranges of W1, W2 and W3. The total mass of the feed was 1.445, so the range of values for the − 10 μm powder mass, W1, is [0,1.445].

From geometry, we know that a straight line has one dimension, while a flat shape such as a square, circle or ellipse has two, and a cube or ball has three. For everyday objects, the dimensions range between one and three, so the range of the fractal dimension of the particle size distribution, W2, is [1, 3].

For *d*_97_, the lower limit of the domain is 0 while the upper limit is equal to the maximum particle size in the raw material (130.37), such that W3 is [0,130.37].

(3) Selecting the affiliation function. Traditionally, an affiliation function is selected to appropriately reflect the parameters according to their properties. According to the experimental kinetic model curve, the ultrafine grinding process of the ball mill becomes increasingly difficult as the process proceeds. In this paper, a *k*-times parabolic affiliation function was selected, with *k* = 2 and the values of *a* (lower limit) and *b* (upper limit) varying for different fuzzy processing objects.

Indicator W1 is a benefit-type indicator (larger values are better), whereas W2 and W3 are both cost-type indicators (smaller values are better). As such, these three indicators require slightly different affiliation functions. The following formulae—Eqs. (3), (4) and (5)—are used as the affiliation functions for W1, W2 and W3, respectively.

3 $$f\left(x;{0,1.445,2}\right)=\left\{\begin{array}{l}0,x\le 0\\ {(\frac{x}{1.445})}^{2},0<x<1.445\\ 1,x\ge 1.445\end{array}\right.$$

4 $$f\left(x;{1,3},2\right)=\left\{\begin{array}{l}1,\text{x}\le 1\\ {(\frac{3-\text{x}}{2})}^{2},1<\text{x}<3\\ 1,\text{x}\ge 3\end{array}\right.$$

5 $$f\left(x;{0,130.37,2}\right)=\left\{\begin{array}{l}1,x\le 0\\ {(\frac{130.37-x}{130.37})}^{2},0<x<130.37\\ 1,x\ge 130.37\end{array}\right.$$

(4) Fuzzy transformation of indicators. The values of each indicator of the test results were fuzzily transformed into the affiliation degree *μ*, which was normalized to [0,1] by the affiliation degree function. Taking the indicators of the product in the blank tests as examples, the fuzzy transformation of indicators was calculated as follows.

The product of the − 10 μm material mass, W1, was 1.31 kg and, since 0 < 1.31 < 1.445, the degree of membership can be obtained by substitution into the subordinate degree function calculation, given by

$$P1=f(1.31;0,{1.445,2})={\left(\frac{1.31}{1.445}\right)}^{2}=0.82.$$

The degree of membership *P*2 of W2 can be calculated as

$$P2=f\left(2.54;{1,3},2\right)={\left(\frac{3-2.54}{2}\right)}^{2}=0.05.$$

The degree of membership *P*3 of W3 can be calculated as

$$P3=f\left(14.27;{0,130.37,2}\right)={\left(\frac{130.37-14.27}{130.37}\right)}^{2}=0.79.$$

The fuzzy evaluation set of each indicator of this test result is, therefore, given by *P* = (0.82,0.05,0.79).

(5) Comprehensive evaluation of multiple indicators. The comprehensive evaluation of multiple indicators is the product of the fuzzy evaluation set of individual indicators and the matrix of the weight distribution set of each indicator, given by

$$Z={P}_{1\times 3}\cdot {W}_{3\times 2}=\left({0.82,0.05,0.79}\right)\cdot {\left({0.69,0.149,0.161}\right)}^{T}=0.70$$

Finally, the average value of the fuzzy comprehensive evaluation index was calculated to explore the influence of roasting pre-cracking on the products. The comprehensive evaluation values (*Z*1, *Z*2 and *Z*3) calculated from the three experimental data of the blank test products are shown as follows.

$$Z1=0.70,$$

$$Z2=\left(0.80, {0.06,0.76}\right)\cdot {\left({0.69,0.149,0.161}\right)}^{T}=0.68,$$

$$Z3=(0.89,{0.04,0.81})\cdot {({0.69,0.149,0.161})}^{T}=0.75.$$

**Figure 8:**
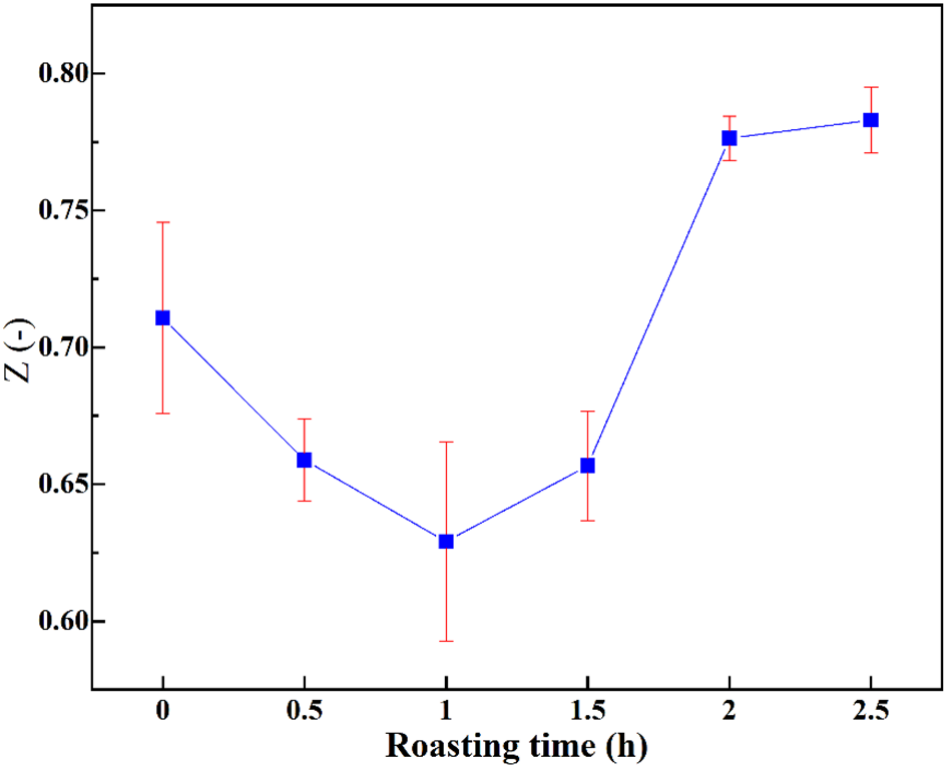
Fuzzy comprehensive evaluation values of different processing modes.

So, the mean comprehensive evaluation index of the blank test products is 0.71 with a standard deviation of 0.03. The comprehensive evaluation indexes calculated from the data from the three experiments where ultrafine products were roasted for different times are shown in Fig. 8.

The means and standard deviations were obtained and a comparison of the comprehensive evaluation indicators shows that the ultrafine potash-bearing shale after roasting pre-cracking was not always superior to the direct ultrafine pulverised product. Roasting for 2 h or 2.5 h contributed 0.07 to the comprehensive evaluation value of the ground product. This has positive implications for the use of potassium-bearing shale for processing into potassium-bearing fertilizers.

### Effect of roasting pre-cracking on comminution kinetics

The concept of kinetic breakage has been described by Austin et al.^24^. Based on the *m*-order comminution kinetic model that has been accepted by the comminution community^25^, *d*_max_ is given by Eq. (6).

6 $${d}_{max}\left(t\right)={d}_{0}\text{exp}(-g{t}^{m})$$

The kinetic curve was fitted using the Levenberg–Marquardt algorithm, where *t* is time (min), *d*_0_ is the particle size of the largest particle in the feed material (μm), *d*_max_ is the particle size of the largest particle in the product after a comminution time *t* (μm), *g* is a parameter related to the size of the material and *m* is a parameter related to the nature of the material.

The kinetic equations of comminution after roasting pre-cracking for different times were obtained, and the model parameters and correlation coefficients are shown in Table 3.

**Table 3:** Parameters related to the comminution kinetics of shale roasted for different times.

Roasting time (h)	*g*	*m*	*R*^2^
0	0.09	0.55	0.98
0.5	0.12	0.47	0.97
1	0.14	0.44	0.99
1.5	0.16	0.42	0.99
2	0.07	0.59	0.99
2.5	0.04	0.69	0.99

Short-time roasting pre-cracking will make the *g*-value increase gradually, indicating that the comminution velocity increases. Roasting for 2 h or more will make the *g*-value decrease, indicating that the comminution velocity decreases^26,27^.

### Economic feasibility analysis

The previous analysis shows that roasting pre-cracking of potassium-bearing shale for 2–2.5 h can improve the fuzzy comprehensive evaluation index of the product; however, industrial applications also need to consider the economic applicability. Compared to the blank test, the roasting pre-cracking process mainly involves higher electricity and equipment depreciation costs. The cost consumption is calculated as 360 days per year at ¥0.9 per kWh of electricity. The intelligent fibre chamber resistance furnace cost ¥4300 and the ball mill cost ¥5,000, and both have a life expectancy of 15 years. For the blank test, the cost is calculated as 1 kW × 3 h × 0.9 RMB/kWh + (3 h/(15 a × 360 d × 24 h)) × 5000 RMB = 2.82 RMB. With roasting pre-cracking for 2 h, the cost is calculated to be 4 kW × 2 h × 0.9 RMB/kWh + (2 h/(15 a × 360 d × 24 h)) × 4300 RMB + 1 kW × 3 h × 0.9 RMB/kWh + (3 h/(15 a × 360 d × 24 h)) × 5000 RMB = 10.08 RMB.

The fuzzy comprehensive evaluation value increased from 0.71 to 0.78, while the cost consumption increased from 2.82 RMB to 10.08 RMB. It is clear that roasting pre-cracking of potassium-bearing shale will not be feasible in industrial applications unless its benefits are proportional to its costs.

## Conclusions

The particle size distributions of both the raw potassium-bearing shale and the ultrafine product crushed by the high-energy ball mill conformed to the RRB particle size distribution model, with a correlation coefficient of 0.99. Compared to the experimental product without any pre-treatment, roasting pre-cracking for 0.5–1.5 h did not reduce the particle size of the product, while roasting pre-cracking for 2–2.5 h did. Roasting pre-cracking caused the particle size distribution to narrow. The fractal dimension of the particle size distribution of the products decreased and then increased slightly. The AHP-fuzzy comprehensive evaluation model, which takes into account both qualitative and quantitative parameters, shows that roasting pre-cracking for 2–2.5 h is effective. Roasting for 0.5–1.5 h may only increase the hardness of the product, which is not conducive to ultrafine pulverisation. Roasting pre-cracking for 2–2.5 h can cause particles to expand into internal cracks, develop new cracks, or increase existing ones, thus loosening the physical framework of the material and making it more brittle, more efficiently crushed and of better quality. Roasting pre-cracking for 2 h, however, greatly increases the cost outlay, which is not favourable for industrial production.
